# Humeral rotational osteotomy for malunion after intramedullary nailing in humeral shaft fracture: a case report

**DOI:** 10.1016/j.xrrt.2024.04.003

**Published:** 2024-04-23

**Authors:** Ryosuke Tsujisaka, Noboru Matsumura, Yusaku Kamata, Hideo Morioka, Yasuhiro Kiyota, Taku Suzuki, Takuji Iwamoto

**Affiliations:** a Department of Orthopedic Surgery, Keio University School of Medicine, Tokyo, Japan; b Department of Orthopedic Surgery, National Hospital Organization Tokyo Medical Center, Tokyo, Japan

**Keywords:** Humeral shaft fracture, Malunion, Rotational deformity, Rotational osteotomy, Corrective osteotomy, Intramedullary nailing, Osteosynthesis

Humeral shaft fractures account for 1%-3% of all fractures.^6^ Although humeral shaft fractures are often treated surgically using an intramedullary nail or locking plate with the minimally invasive plate osteosynthesis technique, rotational malunion has been reported as a postoperative complication of closed reduction. Malrotation exceeding 20° was documented in 27.2% following intramedullary nailing^3^ and in 40.9% after application of the minimally invasive plate osteosynthesis technique.^7^ A rotational deformity of more than 20° is reported to cause shoulder dysfunction and a high rate of secondary arthropathy.^3^ Herein, we report a case of shoulder dysfunction due to rotational malunion after intramedullary nailing in a humeral shaft fracture that was successfully treated by corrective osteotomy of the humeral shaft.

The patient was informed that data concerning this case would be submitted for publication and provided consent.

## Case report

**Figure 1:**
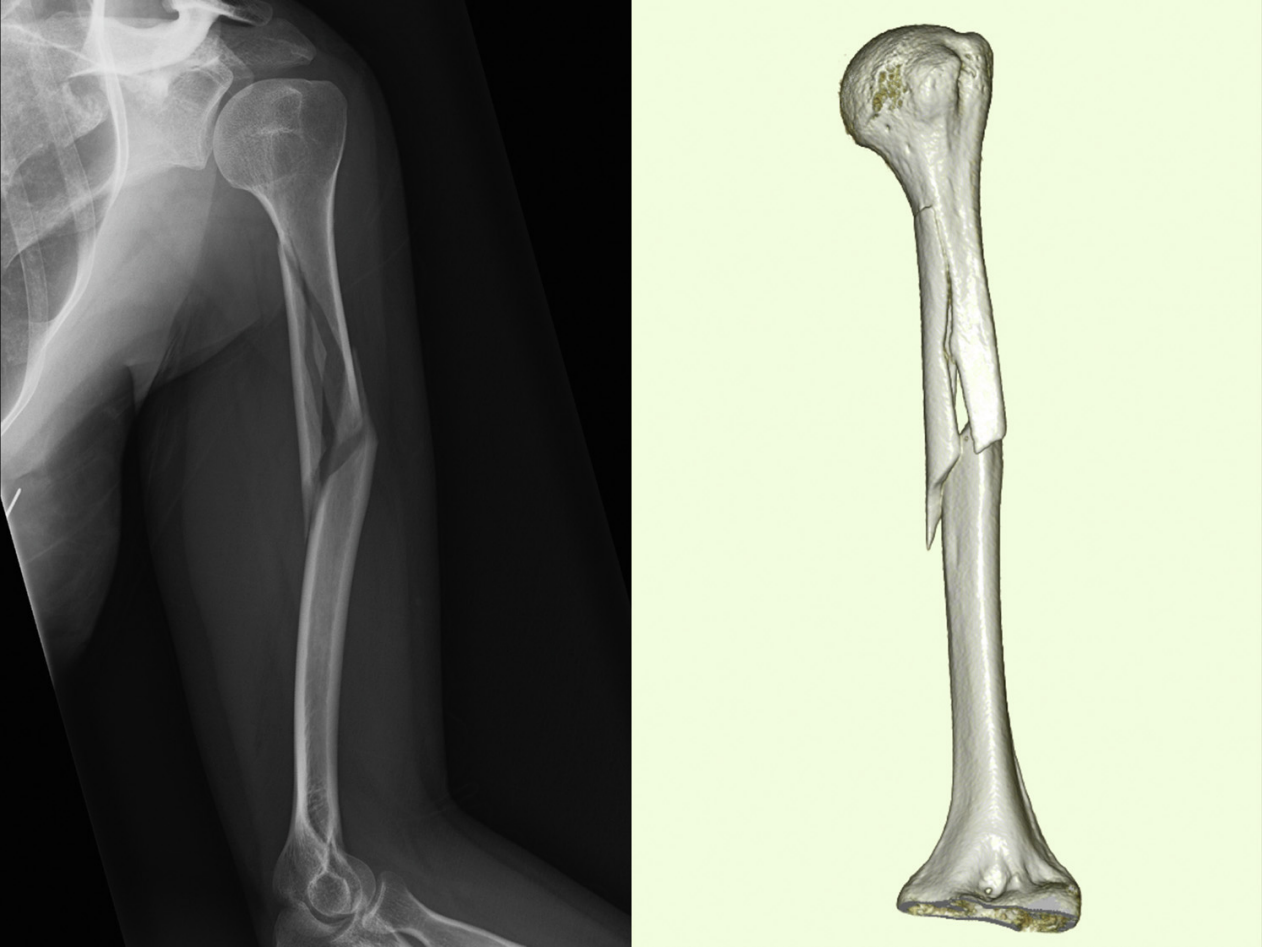
Plain radiographs and CT scans showing the oblique multifragmentary fracture of the left humeral shaft. *CT*, computed tomography.

**Figure 2:**
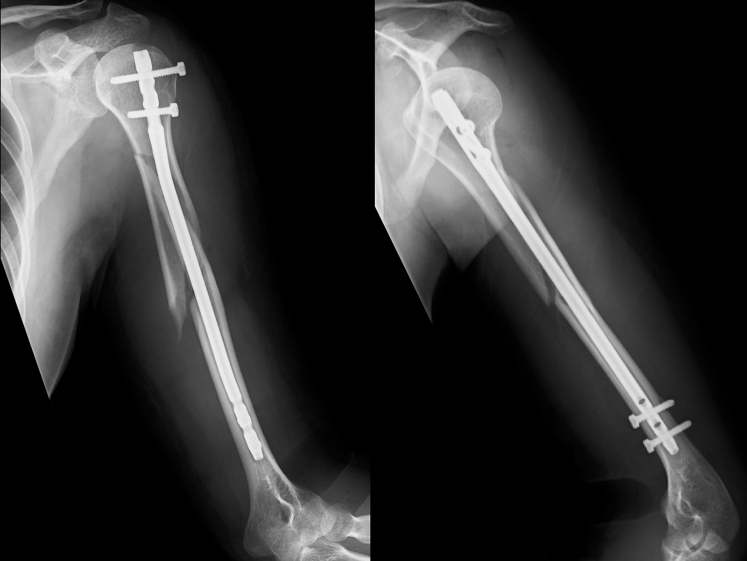
Plain radiographs after osteosynthesis.

A 43-year-old right-handed female sustained a humeral fracture after a fall. Plain radiography showed an oblique multifragmentary fracture of the left humeral shaft. The deltoid tuberosity was attached to the proximal bone fragment. Computed tomography (CT) at the time of injury showed that the deltoid muscle was mainly attached to the proximal bony fragments, the medial head of the triceps brachii was mainly attached to the third bony fragment, and the brachioradialis muscle was attached to the distal bony fragment (Fig. 1). As the patient was highly active and hoped for a quick return to daily activities, the attending doctor performed osteosynthesis 5 days after injury. Through a 5-cm incision on the anterolateral side of the left shoulder, an antegrade interlocking nail (T2 humeral nail; Stryker, Kalamazoo, MI, USA) with a diameter of 7 mm was inserted (Fig. 2).

**Figure 3:**
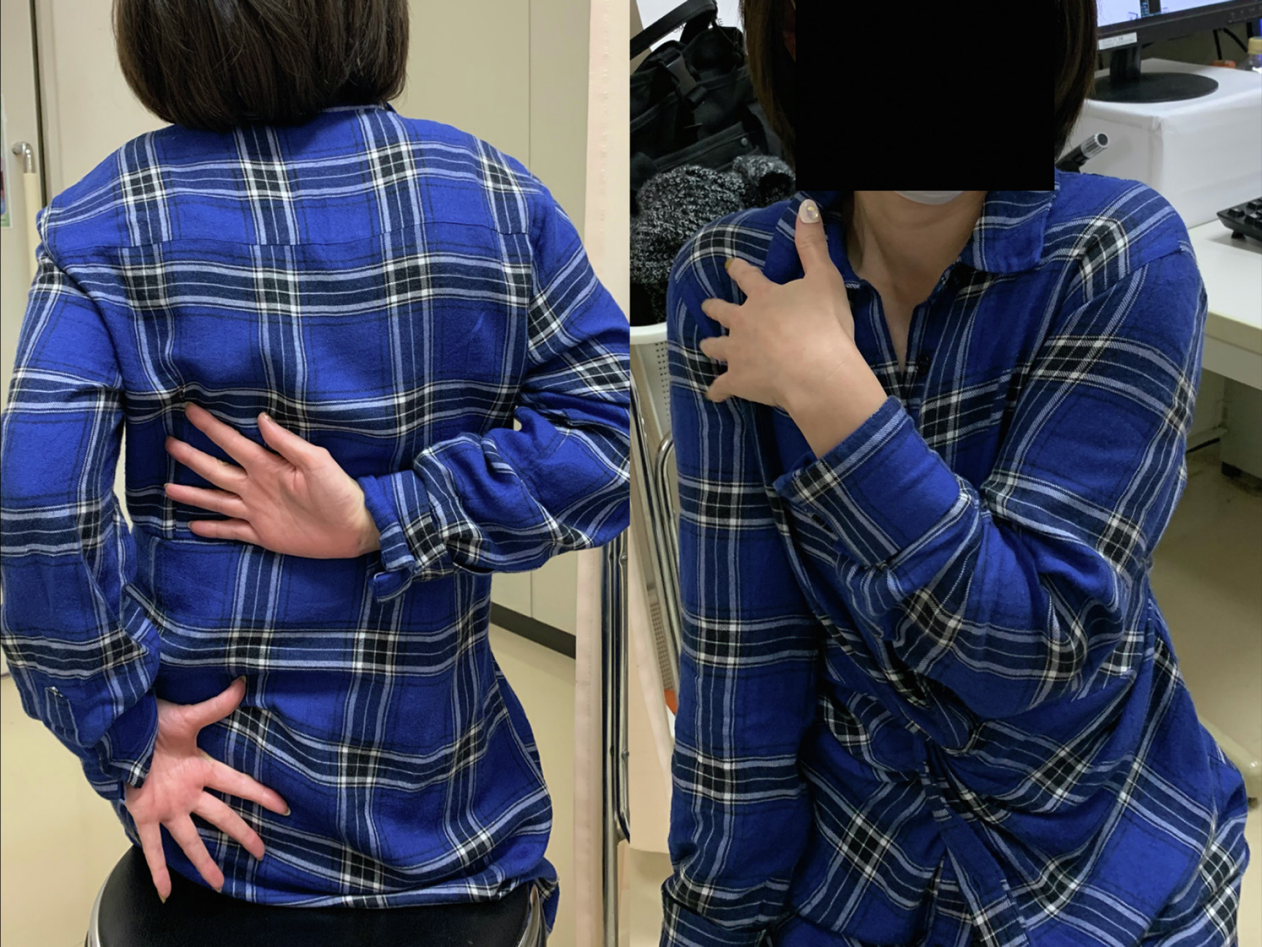
The patient’s limited shoulder range of motion 8 months after osteosynthesis.

**Figure 4:**
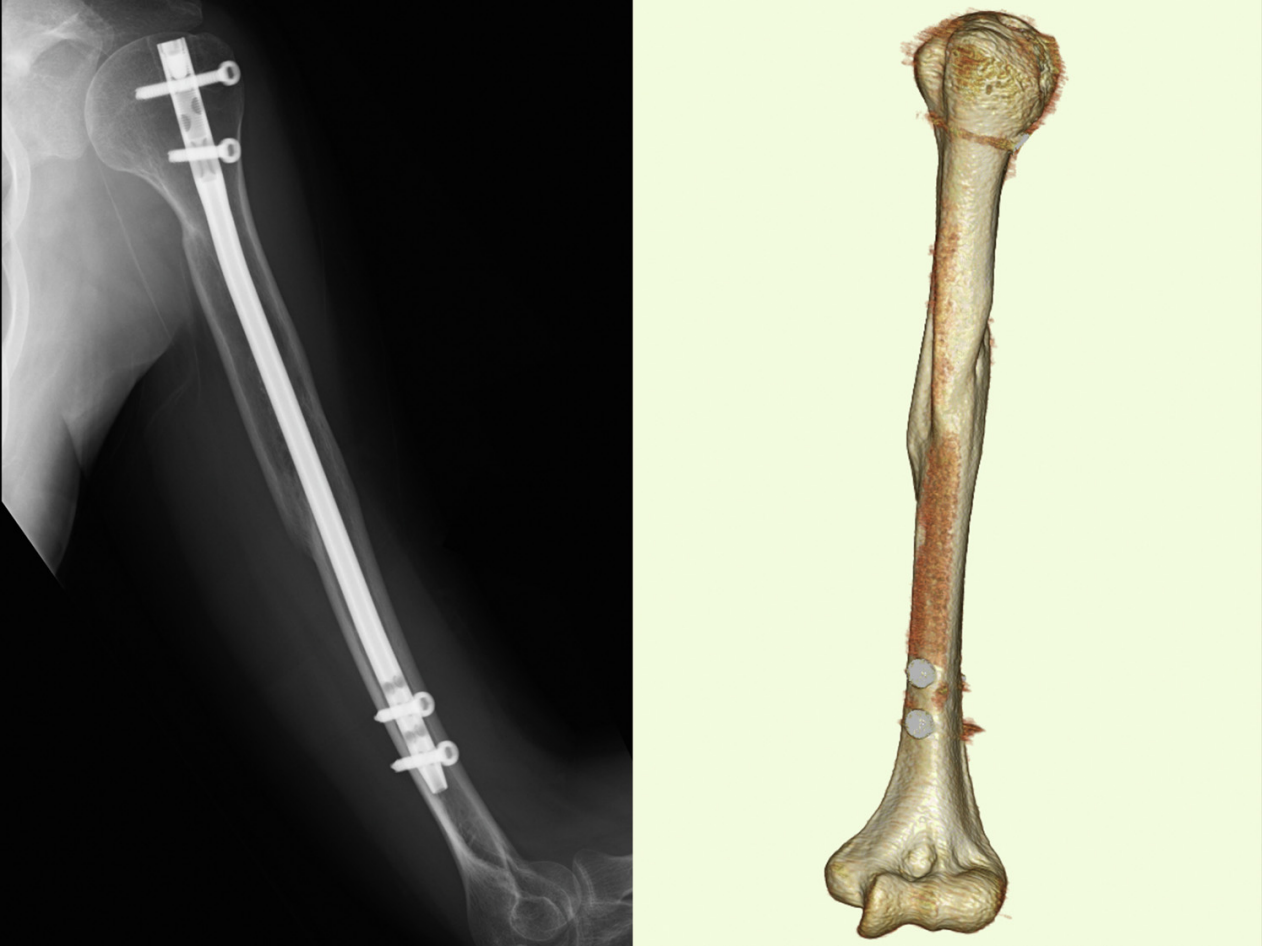
Plain radiographs and CT scans 8 months after surgery showing rotational deformity with 118° of humeral retroversion between the humoral head and elbow axis. *CT*, computed tomography.

However, regardless of successful bone union 6 months after surgery, the patient’s shoulder showed a severely limited range of motion (ROM) compared with the contralateral side, and she had difficulty performing daily tasks with shoulder internal rotation, such as washing her body and putting on and taking off clothes (Fig. 3). She was referred to our institution for discussion regarding options to relieve her symptoms. Six months after surgery, active and passive shoulder motion was 130° in anterior elevation, 90° in external rotation with the arm at the side, and the third lumbar vertebral level in internal rotation behind the back on the affected side; whereas it was 175° in anterior elevation, 90° in external rotation with the arm at the side, and the sixth thoracic vertebral level in internal rotation behind the back on the contralateral side. CT scans taken 8 months after surgery showed a rotational deformity of the left humerus. The degree of humeral retroversion was calculated using CT scans, which is reported to correlate well between sides,^4^ and was 58° on the unaffected side and 118° on the affected side (Fig. 4). Compared with the right humerus, the distal fragment of the left humerus was externally rotated with respect to both the proximal and third fragments. As the most proximal interlocking screw was prominent under the skin, we regarded it might cause a limited ROM by impinging upon the acromion. Consequently, it was extracted 8 months after surgery, but the patient’s shoulder ROM did not improve. Hence, since a humeral rotational deformity of 60° was thought to cause limitation of ROM and functional disability, correction of rotational malunion was planned.

**Figure 5:**
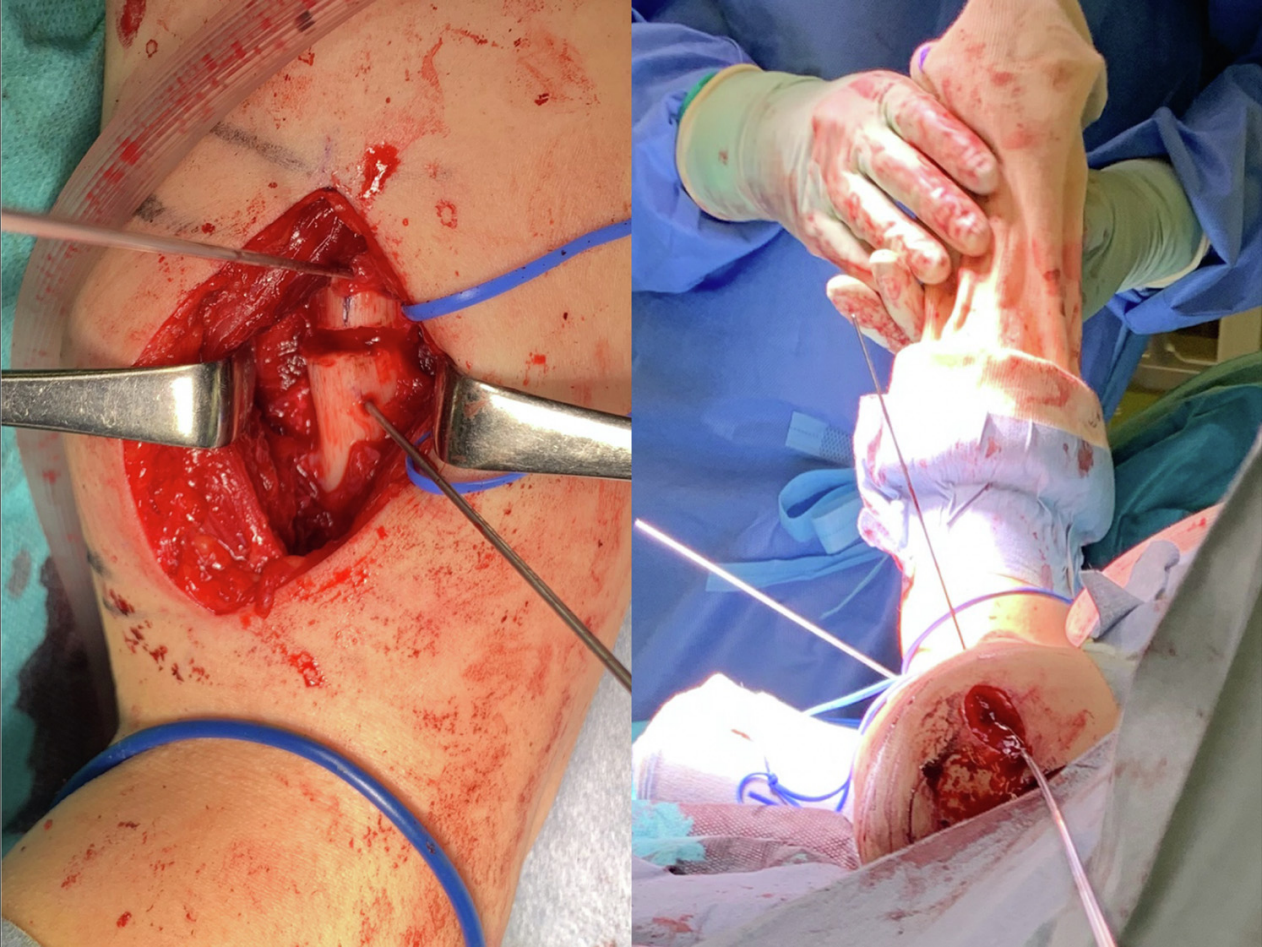
During corrective osteotomy, 2 1.5-mm threaded Kirschner wires are placed 1-cm proximal and 1-cm distal to the site of the osteotomy to assist the correction of the 60° humeral malrotation.

**Figure 6:**
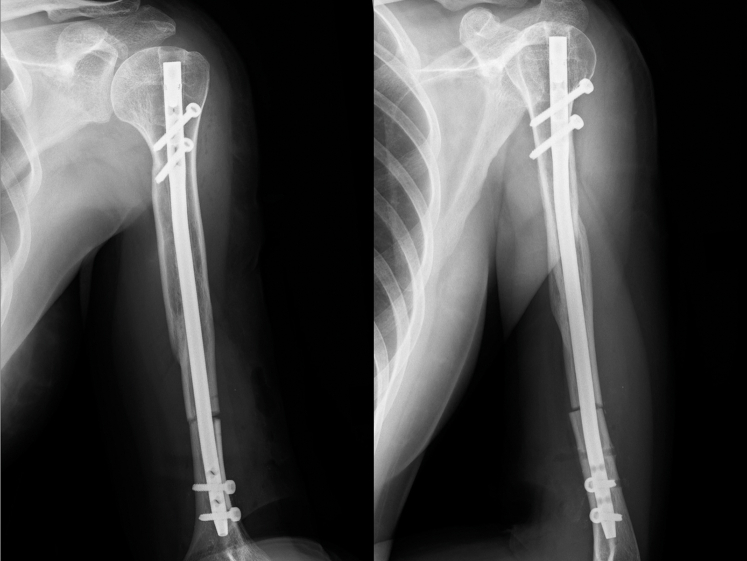
Plain radiographs after humeral rotational osteotomy.

Humeral rotational osteotomy was performed 13 months after the initial surgery. The patient was placed in the beach chair position. A deltoid-splitting approach was used through the same incision as the previous surgery. After the middle portion of the deltoid muscle was bluntly split and the supraspinatus muscle was divided through its fibers, the proximal and distal screws and the intramedullary nail were removed. Then, the intramedullary canal was reamed 10-mm distal to the distal end of the nail using a reamer with a diameter of 8 mm. A 6-cm incision was made on the anterolateral side of the distal third of the upper arm. The biceps brachii muscle was retracted medially and the radial nerve was identified on the brachialis muscle. The brachialis muscle was split to expose the humeral shaft. Given the external rotation of the distal part of the humerus relative to both the proximal and third fragments at the fracture onset, the site of transverse osteotomy was determined to be the distal third of the humeral shaft between the deltoid insertion and the origin of the brachioradialis muscle. The correction angle was set at 60°, as seen in the CT measurements. To measure the amount of rotation, 2 1.5-mm threaded Kirschner wires were monocortically placed 1-cm proximal and 1-cm distal to the planned osteotomy site in parallel. After 2 Homan retractors were placed around the humerus to protect the radial nerve, transverse humeral osteotomy was performed using a 1.2-mm Kirschner wire and an osteotome. Subsequently, a 220-mm intramedullary nail, whose length was the same as that used in the initial surgery, was inserted up to a depth of 10 mm from the top of the proximal humerus. After insertion of two proximal interlocking screws, the distal fragment of the humeral shaft was internally rotated 60° from the previous position with the assistance of Kirschner wires (Fig. 5), and 2 distal transverse screws were inserted using a radiolucent drill. After fixation, the split muscles were repaired and the wound was closed (Fig. 6).

**Figure 7:**
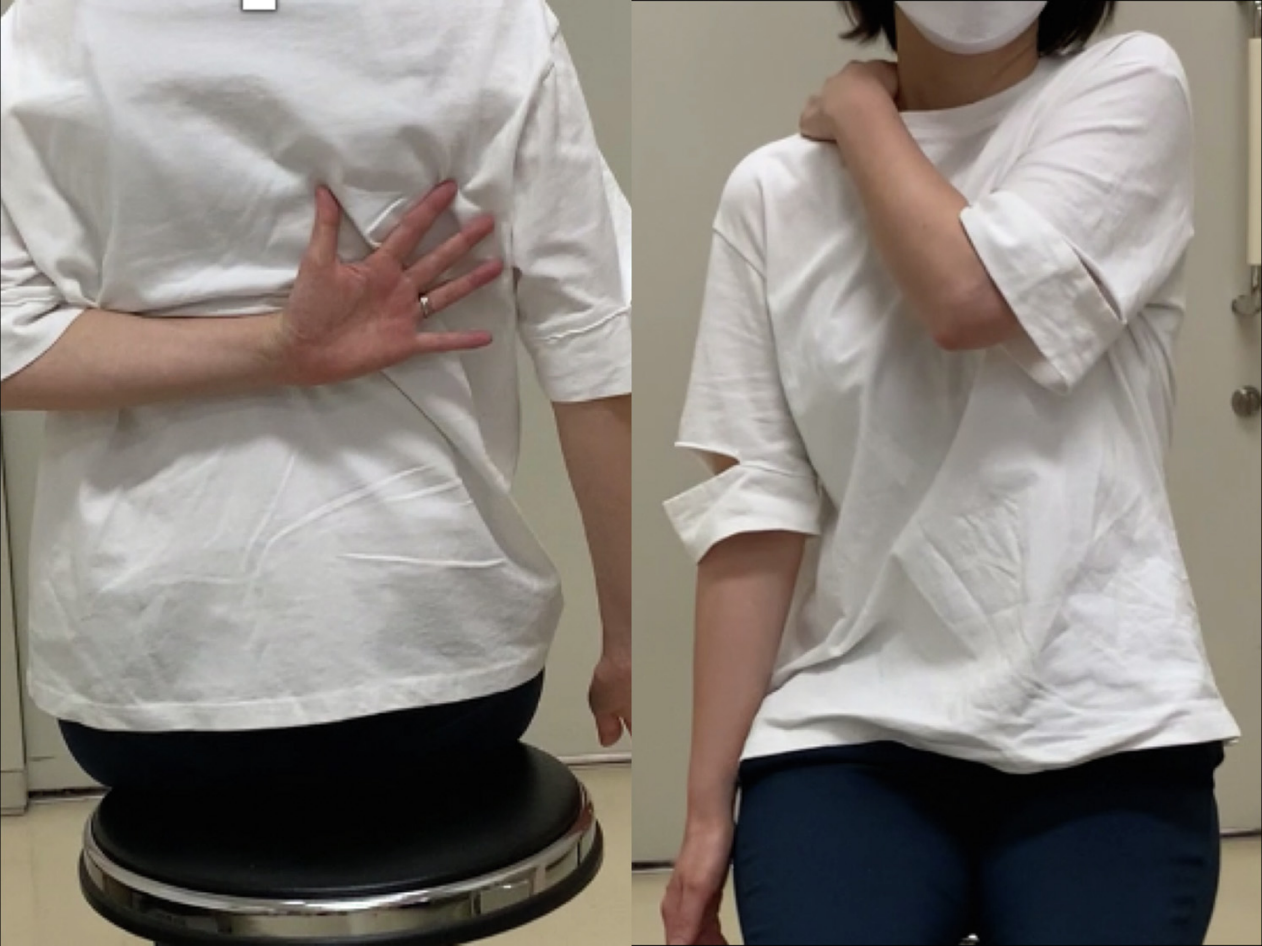
The patient’s shoulder range of motion 1 year after corrective osteotomy.

The arm was placed in the sling position after corrective osteotomy. Stooping, pendulum, and passive shoulder ROM exercises were started a week after surgery. Active shoulder motion was allowed at 2 months postoperatively. Sports activity, heavy work, and weight-bearing were allowed 4 months after surgery after bone union was confirmed on plain radiographs. At the final follow-up, which was 1 year after corrective osteotomy of the humerus, successful bone union was achieved on plain radiographs and CT scans. Humeral retroversion was 54° on CT scans. The patient was able to use her upper extremity for internal rotation without any complications. The shoulder ROM recovered to 170° of elevation and the seventh thoracic vertebral level in internal rotation behind the back, while external rotation on the affected side decreased to 70° (Fig. 7). The American Shoulder and Elbow Surgeons Shoulder scores improved from 73 points before corrective osteotomy to 91 points at the final follow-up.

## Discussion

We described a case of iatrogenic rotational malunion of the humerus after intramedullary nailing that was successfully treated with humeral rotational osteotomy. In this case, a 60° rotational deformity occurred compared with the contralateral humerus. The reason for the malrotation was assumed that the arm was placed with excessive external rotation when the screws were inserted to the distal fragment. The present case showed an oblique multifragmentary fracture of the humeral shaft, which made it difficult to evaluate the rotational deformity of the humeral shaft under the fluoroscope at the time of the initial injury. To avoid rotational malalignment, close attention should be paid to the limb position of the distal humerus during screw insertion.

As the patient’s clinical symptoms were thought to be due to a rotational deformity, osteotomy to correct the rotational deformity was performed. The anatomical muscle attachment site should be considered when performing corrective osteotomies. In previous reports of the sequelae of brachial plexus birth injuries or locked shoulder dislocations,^5^^,^^8^^,^^9^ the majority of rotational osteotomies have been performed at a level proximal to the insertion of the deltoid and rarely been performed distal to the deltoid attachments. In the present case, however, CT scans at the time of injury showed that the deltoid was mainly attached to the proximal fragment and the brachioradialis was attached to the distal fragment, and CT scans before corrective osteotomy revealed that the distal fragment was externally rotated at a level below the deltoid tuberosity. Considering the original anatomical position, we regarded that the positional relationship between the deltoid muscle and the deltoid tuberosity should not be altered. Therefore, corrective osteotomy was performed in the distal one-third of the humeral shaft between the deltoid insertion and origin of the brachioradialis muscles.

Several reports have described plate fixation after osteotomy of brachial plexus palsy.^1^^,^^2^ Compared with plate fixation, intramedullary nail fixation might be less accurate to correct the deformity in cases with fracture malunion. However, in the present case, an intramedullary nail was inserted during the initial surgery. Thus, reinsertion of an intramedullary nail deeper than the initial surgery was performed instead of plate fixation, which requires additional invasion to the soft tissues. With the assistance of monocortically placed Kirschner wires, intraoperative assessment of the corrective angle is relatively easy during nailing. To the best of our knowledge, no report has shown either corrective osteotomy of the humerus using an intramedullary nail or exchange nailing for the management of humeral rotational deformity after intramedullary nailing. The patient’s ROM and shoulder dysfunction successfully improved after osteotomy without any complications, hence, we suppose that humeral rotational osteotomy with nail reinsertion is a viable and useful treatment option for iatrogenic malunion after osteosynthesis using an intramedullary nail.

## Conclusion

We report a case of shoulder dysfunction due to rotational malunion after intramedullary nailing in a humeral shaft fracture that was successfully treated by corrective osteotomy of the humeral shaft. The reason for malunion was assumed that the arm was placed with excessive external rotation when the screws were inserted to the distal fragment. Close attention should be paid to the limb position of the distal humerus during nailing. Humeral rotational osteotomy with nail reinsertion is a viable and useful treatment option for iatrogenic malunion following osteosynthesis using an intramedullary nail.

## Disclaimers:

Funding: No funding was disclosed by the authors.

Conflicts of interest: The authors, their immediate families, and any research foundations with which they are affiliated have not received any financial payments or other benefits from any commercial entity related to the subject of this article.

Patient Consent: Obtained.
